# The Impacts of Educational Asthma Interventions in Schools: A Systematic Review of the Literature

**DOI:** 10.1155/2016/8476206

**Published:** 2016-08-30

**Authors:** Ana Carla Carvalho Coelho, Laís Souza Barretto Cardoso, Carolina de Souza-Machado, Adelmir Souza-Machado

**Affiliations:** ^1^Escola de Enfermagem, Universidade Federal da Bahia, Salvador, BA, Brazil; ^2^ProAR, Programa para o Controle da Asma na Bahia, Universidade Federal da Bahia, Salvador, BA, Brazil; ^3^Programa de Pós-Graduação em Medicina e Saúde, Faculdade de Medicina da Bahia, Universidade Federal da Bahia, Salvador, BA, Brazil; ^4^Instituto de Ciências da Saúde, Universidade Federal da Bahia, Salvador, BA, Brazil

## Abstract

*Objective*. To review the literature on the impact of educational asthma interventions in schools regarding the knowledge and morbidity of the disease among children and adolescents.* Methods*. A systematic review was conducted for controlled clinical trials investigating the effectiveness of educational asthma interventions for students, asthmatic or nonasthmatic, families, and school staff. Databases were CENTRAL, PubMed, LILACS, MEDLINE, and SciELO. Articles published in any language were considered, in the period from 2005 to 2014, according to the PRISMA guidelines.* Results*. Seventeen articles were selected (*N* = 5,879 subjects). 94% of the interventions (16 of 17 studies) were applied in developed countries that were led by health professionals and most of them targeted asthmatics. Asthma education promotes the improvement of knowledge about the disease in at least one of the evaluated areas. 29% of the interventions (5 of 17 studies) showed a reduction of the asthma symptoms, 35% (6 of 17 studies) reduction of the hospitalization instances and emergency visits, 29% (5 of 17 studies) reduction of school absenteeism, and 41% (7 of 17 studies) increase in the quality of life of the individuals.* Conclusions*. Educational interventions in schools raise the awareness of asthma and weaken the impact of morbidity indicators.

## 1. Introduction

Asthma affects approximately 334 million individuals and is the 14th highest disease around the world in terms of duration and prevalence of disability [1]. In Brazil, it is estimated that 20% of school-age individuals are asthmatic [2, 3], which is one of the highest prevalence rates in the world. In addition, asthma is the most common chronic respiratory disease in pediatric age groups [2] and is responsible for profound negative social and economic impacts which are associated with the worsening of the disease [1].

As with other chronic diseases, asthma affects the performance of children and adolescents at home and school. Among individuals in this age group, the negative impact of asthma drives school absenteeism, social isolation, and reduced quality of life [4, 5]. This fact could be associated with lack of information on the disease and ignorance of the disease among patients, families, health professionals [6], and school staff [7, 8] who might not recognize asthma as a potentially serious illness and may underestimate the symptoms of the disease.

Access to information, increased knowledge, the acquisition of new behaviors and habits, and the improvement of the health status of the population are the main objectives of health education activities, particularly those regarding asthma in schools [9]. For much of the population, these activities are the only means of access to systematic knowledge about health [10]. In regard to asthma, these actions are able to identify students with suggestive symptoms of asthma [9, 11, 12] and teach actions to manage the disease [9, 13–17]. Thus, activities in schools can be a simple alternative to reduce the level of morbidity [16, 18] and improve the quality of life [19] related to asthma in the school community and the wider community around the school center [15].

The difficulties in the management of asthma in schools, such as the lack of communication between students, families, and school staff and the lack of training of teachers and school staff to recognize possible cases of asthma and provide the care needed in this environment, should be considered [15].

Thus, there is a need to spread awareness of asthma in the whole school community [20–22], regardless of a previous diagnosis of asthma [20, 23]. Common knowledge of a serious public health problem among the masses can positively impact early recognition of cases that are suggestive of asthma and the initial management of asthmatics in the school environment when necessary [9, 11].

The aim of this study was to systematically review the literature on the impact of educational interventions on asthma which are held in the school environment for an understanding of the knowledge of the disease among students, asthmatic or nonasthmatic, members of the school community, and the main outcomes on morbidity among children and adolescents with asthma.

## 2. Materials and Methods

### 2.1. Study Design

The present study is a systematic review of controlled clinical trials, both randomized and nonrandomized, which investigated the efficacy of educational asthma interventions that were carried out in a school environment.

### 2.2. Sources of Information

The search for the studies was conducted using 3 electronic databases of the leading publications in the biomedical literature, namely, the Cochrane Central Register of Controlled Trials (CENTRAL), PubMed/Medical Literature Analysis and Retrieval System Online (MEDLINE), and the BIREME Virtual Health Library (LILACS, MEDLINE, and SciELO). We considered studies that were not selected by the search strategy but were identified in the references of the selected articles that met the eligibility criteria or in the references of the published guidelines used in this review. Data were collected from October 2014 to August 2015.

### 2.3. Search Strategies

Studies were selected after defining the DeCS and MeSH search terms, such as asthma/asma, children/crianças, adolescent/adolescente, schools/escolas, health/saúde, and intervention study/estudo de intervenção. These terms were crossed via Boolean switch statements (AND), as shown in the following topics: (i) asthma and children and schools and health; (ii) asthma and children and schools and health and intervention study; (iii) asthma and adolescent and schools and health; (iv) asthma and adolescent and schools and health and intervention study.

Articles in any language published in the 2005–2014 period were considered. These years of searching were chosen because, during this period, guidelines and public policies that supported the construction of structured educational programs on health and asthma in schools were published internationally, namely, the National Asthma Education and Prevention Program (NAEPP) [24], Students with Chronic Illnesses: Guidance for Families, Schools and Students [25], and a public policy guide for the management of asthma in schools by the education team of the state of Michigan, USA [26].

Studies were included which discussed actions, interventions, and education programs for asthma which were conducted in rural or urban school environments in public or private schools and aimed at students, asthmatics and nonasthmatics, parents, and school staff of primary education.

### 2.4. Selection of the Studies

One author (A1) was responsible for the collection of studies in databases according to the search strategy defined earlier. After the exclusion of duplicates, the titles and abstracts of the studies were read to exclude those that did not meet the inclusion criteria. All of the selected articles were read in full, according to the established criteria, and organized and independently reviewed by another author (A2). The inclusion and exclusion criteria are described as follows.

*Inclusion Criteria and Exclusion of Articles*

*Inclusion Criteria*

1. The articles must investigate the effectiveness and/or efficiency of educational asthma interventions conducted in the school environment.
2. Studies must be designed as controlled clinical trials, randomized or not, with a minimum duration of one month.
3. Articles should have a target audience of students of both genders, aged between 10 and 19 years, asthmatics or with symptoms suggestive of asthma. Articles may also have a target audience of nonasthmatic individuals, parents or caregivers, and staff.
4. Studies may be published in any language in the last 10 years.

*Exclusion Criteria*

1. Studies should not evaluate educational asthma interventions outside of the school environment or involve the efficacy of new drugs.
2. Studies should not lack primary or secondary outcomes (described in Primary and Secondary Outcomes Analyzed in the Selected Articles).
3. Studies should not have an adult-only target audience of the interventions.
4. Duplicate or complimentary studies were omitted.

### 2.5. The Data Collection Process

The articles that were selected and reviewed by A1 were also reviewed by A2 and discussed among peers for standardization of information. In case of disagreements regarding each topic to be analyzed in the study, a third author (A3) was responsible for solving the disagreement and a final evaluation.

For each included article, the following variables were identified: (1) location of the study; year of publication; (2) study design, sample size, and age group; (3) applied educational strategy, amount of educational sessions, and duration of each session; (4) the approach used (individual, group, or mixed); (5) an indication of whether there was curricular integration; and (6) the primary and secondary outcomes evaluated in the study period which were understood as the time between the initial period (preintervention) and the final evaluation of the intervention. The primary and secondary outcomes evaluated are listed as follows.

*Primary and Secondary Outcomes Analyzed in the Selected Articles*

*Primary Outcomes*. Primary outcomes include knowledge of asthma (concept, pathophysiology, triggers, treatment, action plan, and beliefs about asthma).

*Secondary Outcomes (Clinical). *Secondary outcomes include the following:

1. Signs and symptoms of asthma and exacerbation, independent of severity
2. Hospitalization instances due to asthma
3. Visits to an emergency department due to exacerbation of asthma
4. Quality of life
5. Number of days absent from school
6. Use of relief and control medications
7. Use of the action plan.

## 3. Results

### 3.1. Selection of the Studies

**Figure 1:**
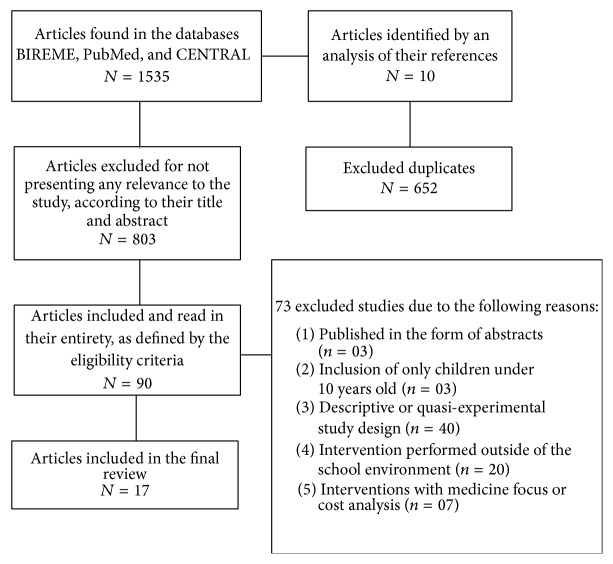
Flowchart of the selection of studies included for a systematic review of the literature.

A total of 1,545 articles were identified; 652 duplicates were excluded. After reading the titles and abstracts, 90 articles were potentially eligible and selected for reading in full, with 73 studies eliminated due to the following reasons: (1) published in the form of abstracts (*n* = 03); (2) inclusion of only children under 10 years old (*n* = 03); (3) descriptive or quasi-experimental study design (*n* = 40); (4) intervention performed outside of the school environment (*n* = 20); and (5) interventions with medicine focus or cost analysis (*n* = 07). In the end, 17 articles were selected according to the eligibility criteria.  Figure 1 shows the process of selecting studies for the systematic review of the literature, according to the Preferred Reporting Items for Systematic Review and Meta-Analyses (PRISMA) guidelines [27]. The points of PRISMA regarding statistical analyses (topics from 12 to 16 and from 19 to 23) were not checked as this is a literature review without meta-analyses.

### 3.2. Characteristics of the Educational Interventions on Asthma

The samples of the studies vary between 30 and 1,292 students and families, totaling 5,879 participants in the 17 articles analyzed. The target population of the interventions varied between studies as follows: (1) asthmatic students [12, 14, 18, 21, 28–33]; (2) parents, caregivers, or family members [16, 17, 19, 22, 30, 34, 35]; (3) school staff [17–20, 30]; (4) individuals without a diagnosis of asthma [17, 20]; and (5) the wider community around the school center [17, 19].

The educational interventions were made up of educational sessions conducted in groups [12, 17–22, 28, 30–32, 34, 35], individuals [29], or mixed samples (groups and individuals) [14, 16, 33]. These sessions lasted 15–120 minutes and were evaluated at intervals ranging from 3 weeks to 24 months. The characteristics of the selected studies and their educational interventions are presented in Table 1.

Issues related to the respiratory system and asthma, such as the concept and pathophysiology [14, 18, 20, 22, 28–33], asthma triggers [12, 14, 16, 18, 20–22, 28–30, 33–35], suggestive symptoms of the disease [12, 14, 16–22, 28, 30–32, 34], relief treatment and maintenance [14, 16, 17, 28, 32, 34, 35], presentation of inhalers [14, 22, 28, 29, 31, 33], asthma self-management [14, 17, 19, 21, 22, 28, 30, 34, 35], the use of the action plan [17, 22, 30, 31], and beliefs on asthma [20], were all discussed in the educational sessions.

The interventions were carried out by health professionals [14, 17–19, 21, 28, 30–33, 35], undergraduate students, community leaders, and previously trained students with primary education [12], health/asthma certified educators [16, 22, 31, 34], trained primary school teachers [20], and an interactive program based on the Internet [29].

Approximately 65% of the studies described their interventions as being integrated into the school day [12, 14, 17–21, 31, 32, 34, 35]. Only one of these interventions was developed in a country with a low median income, which was an Australian model adapted to the cultural and economic needs of Jordan and conducted by peer monitors. Only 17.6% of the studies cited the inclusion of topics related to health in the school curriculum [17, 19, 20]. In these studies, subjects related to the respiratory system and asthma permeated the existing traditional disciplines, such as science, biology, mathematics, and Portuguese, without the addition of new courses in the curriculum [17, 19, 20].

### 3.3. Results Associated with the First Outcome: Knowledge of Asthma in Asthmatic or Nonasthmatic Students, Parents, Caregivers, and School Staff

A low level of prior knowledge of asthma was observed in the assessment of students, asthmatic or nonasthmatic, parents, caregivers, and school staff [16–21, 31, 32]. None of the studies compared the knowledge of asthma among asthmatic and nonasthmatic individuals.

In 8 studies that evaluated 1,974 students, educational interventions were able to improve the level of knowledge of the disease among school-age individuals [16–21, 31, 32]. Of these studies, only 1 was held in a low- or middle-income country [21]. Butz and colleagues [16], in 7 rural schools of the USA, studied 201 asthmatic students and, through an educational intervention on asthma using coloring books, spacers, and peak expiratory flow (PEF) meters, showed that the themes came to be more known by the participants after the intervention, including the example of the anatomy of the respiratory system (control group, 59%, versus intervention group, 86%; *p* = 0.01), the use of PEF (control group, 45%, versus intervention group, 66%; *p* = 0.04), and the use of relief medication (control group, 36%, versus intervention group, 66%; *p* = 0.002). In this same study, the authors found that, among 112 participants, parents, or caregivers in a workshop to guide chiefly for the use of PEF and inhalers, the participants presented a better level of knowledge of the treatment of asthma [16]. No differences were observed between the control group and intervention group regarding the knowledge of the measures for environmental control [16]. Moreover, a satisfactory level of parent knowledge did not positively impact the knowledge of asthmatic individuals about asthma [16].

Mosnaim and colleagues [31] studied 536 asthmatic individuals who participated in an educational intervention on asthma that was carried out during school hours using a focus group and the training on inhalation technique. In this study, after the intervention, increases in knowledge of asthma were observed by as much as 5.7% among adolescents [31]. Pike and colleagues [20] evaluated 236 students, asthmatic or nonasthmatic, and concluded that a curricular intervention may be an alternative to awareness among the school community about asthma [20].

Only in one low-income country was the knowledge of the disease among adolescents with asthma evaluated. The intervention performed in this Jordanian study dealt with the adaptation of a successful program previously conducted in Australia, the “Triple A,” which was conducted by health educators and peer monitors who were previously trained. Al-sheyab and colleagues [21] found an increase in the knowledge of the disease of 13.5% after the intervention.

Improved knowledge of asthma was observed after various school interventions in asthmatics [16–19, 21, 31, 32] or nonasthmatics [20], mainly in high-income countries using group sessions that were led by health professionals and lasted a maximum of 120 minutes. Only 1 intervention was applied in a country of low or medium income. However, in our study, 3 models of curriculum interventions were applicable to low- and middle-income countries such as Chile, Peru, and Kenya. These interventions value the inclusion of issues related to the respiratory system and asthma in the school curriculum aimed at all students, asthmatics or nonasthmatics, and the wider school community [17, 19, 20]. These interventions can be adapted to and/or be conducted by trained school staff without the need for permanent healthcare professionals in the school environment [17, 19, 20].  Table 2 presents the results for the main outcomes evaluated in the trials included.

### 3.4. Results Associated with Secondary Outcomes in Asthmatic Students: Morbidity Indicators

#### 3.4.1. Reduction in the Signs and Symptoms of Asthma

Only 41% of the studies adopted signs and symptoms of asthma as health outcomes. Among the participants of school interventions, there was a reduction in the frequency and intensity of asthma symptoms, mainly of the nocturnal symptoms of asthma. These findings were observed in only 29% of the included studies [14, 16, 29, 33, 35] with samples composed of, for most part, mild and persistent asthmatic adolescents. Based on the study performed by Butz et al. [16], we consider the adolescents attending school from the 3rd to the 5th grades.

Two controlled and randomized clinical trials showed a reduction of asthma symptoms among moderate and severe asthmatic adolescents after participating in interventions that were focused on self-management of the disease [14, 33]. Bruzzese and colleagues [14] studied 345 adolescents with asthma in the USA and identified less nocturnal symptoms related to asthma among students participating in the educational intervention associated with clinical follow-up compared to the control group (intervention group, $\overset{-}{x}=1.42\pm 1.72$, versus control group, $\overset{-}{x}=2.23\pm 2.39$; *p* = 0.001 [14]). Likewise, Joseph and colleagues [33] evaluated 422 adolescents with moderate to severe asthma and observed a reduction in the days with asthma symptoms in the group submitted to an interactive and virtual intervention (intervention group, $\overset{-}{x}=6.2\pm 7.7$, versus control group, $\overset{-}{x}=9.2\pm 8.1$; *p* = 0.013) [33].

Another 2 studies that adopted the signs and symptoms of asthma as an analysis of outcomes observed no reduction in these indicators after intervention [12, 34]. Clark and colleagues [34], in a study conducted in China in 2005 with 639 asthmatic individuals who were mostly intermittent, found no change in symptoms. These authors adopted a tailored intervention program called “Open Airways for Schools” that was composed of interactive group educational sessions led by a health educator [34].

Using the same program, another study in the USA by Clark and colleagues in 2010 found 1,292 asthmatic individuals with a higher proportion of intermittent asthma and also did not observe changes in asthma symptoms after the intervention [12]. These results could be attributed to the presence of intermittent asthmatics that present few symptoms of the disease or symptoms that could be confused with other acute respiratory diseases.

#### 3.4.2. Reduction in Hospitalization Instances and Emergency Department Visits

Seven studies evaluated hospitalization as an outcome and nine studies evaluated visits to emergency rooms. In 57% (4 of 7 studies) of the analyzed studies, educational interventions on asthma reduced hospitalization instances [14, 18, 29, 34]. In 44% (4 of 9 studies), the interventions reduced the emergency department visits due to the exacerbation of the disease [14, 18, 22, 30]. These interventions were conducted by health professionals or health educators and were comprised of sessions of up to 60 minutes long with follow-up periods of 12–24 months.

In 43% (3 of 7 studies), there was no modification of hospitalization instances [16, 17, 33] and in 56% (5 of 9 studies) there was no modification of emergency department visits [16, 17, 29, 33, 34]. Approximately, 18% (3 of 17 studies) of the studies showed no significant reductions in hospitalization instances or visits to emergency departments, simultaneously [16, 17, 33]. This fact can be attributed to the following: (1) carrying out studies in rural environments where health services are more restricted [16]; (2) samples composed of a greater proportion of intermittent or mild persistent asthma cases with fewer reports of healthcare needs [16]; and (3) no classification of the severity of the disease in the study [17].

#### 3.4.3. Increase in the General Quality of Life

Seven of the analyzed studies presented a high overall level of quality of life for students diagnosed with asthma who were participants of interventions [14, 19, 21, 22, 30, 33, 34]. Only 3 studies showed an increase in the median level of all of the areas [14, 21, 30]. The areas evaluated in the study were asthma symptoms, emotional function, physical limitations associated with the disease, and the perception in terms of environmental stimuli at 3 and 12 months after the intervention.

In one of the randomized clinical trials that had an increase in the quality of life, Bruzzese and colleagues [14] achieved this result through an educational asthma intervention that was associated with a clinical follow-up (Table 2). McGhan and colleagues [30], in another randomized trial in Canada, evaluated 266 asthmatic individuals and found similar results through an educational intervention focusing on self-management of asthma.

In 23% of the studies, improvements in quality of life were not observed after educational interventions in asthma [12, 16, 28, 29]. This fact can be attributed to the prevalence of mild asthmatics in the samples of the studies, the unfamiliarity of participants with the quality of life evaluation questionnaires [16, 28], and unattractive interventions for adolescents, which is the least likely age group to adhere to asthma education programs when compared to asthmatic children [12].

#### 3.4.4. Reduction in School Absenteeism

School absenteeism was measured using self-reporting or school records to identify the absence of the participating students. In studies evaluating this outcome, the authors observed a reduction in the number of missed school days among school-age individuals at 6 and 12 months after the intervention, with an average reduction of up to 4.38 days [14, 18, 22, 29, 34]. Moreover, student participants in the control group had twice as many school absences when compared to the participants of the interventions [18].

In one study conducted in 21 schools in industrial and rural areas in China, the authors [34] evaluated 639 individuals aged between 7 and 11 years that participated in a tailored intervention program called “Open Airways for Schools.” A reduction in school absenteeism was found after the intervention and was associated with improved academic performance among the asthmatic students who participated in the control and intervention groups, respectively ($\overset{-}{x}=-0.55$ versus $\overset{-}{x}=-0.32$, *p* = 0.02) [34].

#### 3.4.5. Identification of Undiagnosed Asthma

Publications in the USA that have adopted the same model of educational intervention that was adapted from the program “Open Airways for Schools” identified 2,028 possible cases of asthma in the school environment as part of the intervention using validated questionnaires to detect cases of asthma [12, 17] and a spirometry test for confirmation [17]. This identification of cases that are suggestive of asthma allowed individuals with symptoms of the disease that were yet undiagnosed to have access to educational asthma programs that are able to improve knowledge of the disease, allowing the recognition of symptoms and the necessary measures for self-management.

### 3.5. Use of Relief and Control Medication and Action Plan

The increased use of control medication and the reduction in the need of using relief medication were assessed and measured in 18% of the studies [29, 30, 35]. Joseph and colleagues [29], for example, in a study conducted in the USA with 314 adolescents with asthma who participated in a virtual intervention on asthma found positive behavior and adherence to maintenance medication (intervention group, 20.4%, versus control group, 12.6%; *p* = 0.09) and positive behavior in the evaluation of the use of relief medication (intervention group, 38.8%, versus control group, 32.2%; *p* = 0.01) [29].

At the same time, McGhan et al. [30] in Canada showed an improvement of 127% in the use of relief medication among 266 asthmatic participants in an interactive group intervention, conducted by health professionals. The biggest report of taking relief medicine to the school to use in situations of exacerbation was observed in 23 asthmatic subjects with a mean age of 12.9 who were participants in the program based on “Open Airways for Schools” and “Asthma Self-Management for Adolescents” (intervention group, $\overset{-}{x}=3.0\pm 0.9$, versus control group, $\overset{-}{x}=2.2\pm 0.8$; *p* < 0.05) [35].

A higher frequency of use of the action plan was noted among 345 of the participating students of an educational asthma intervention in 5 schools in the USA. This intervention was associated with a clinical and educational approach for the medical assistants of these adolescents. In this study, students in the intervention group were 4 times more likely to use the action plan at 12 months after the baseline when compared to the control group [14].

## 4. Discussion

In this review, we found that educational actions on asthma conducted in schools raise the level of knowledge of the disease among the participants of the interventions (asthmatic or nonasthmatic). Regarding the morbidity indicators in asthmatics, a small proportion of studies presented educational interventions that were capable of reducing the frequency and intensity of the symptoms of asthma (5 of 17 studies), hospitalization instances and emergency department visits (6 of 17 studies), school absenteeism (5 of 17 studies), and improving the quality of life of individuals (7 of 17 studies).

Few clinical trials with educational asthma interventions included nonasthmatic students [20] and school staff [17–20, 30]. This evidence suggests that models of school education in asthma are designed primarily to search for effective measures to reduce morbidity indicators. However, educational activities for asthma could involve the whole school community, providing opportunities for knowledge of the disease to students, teachers, and school staff [24]. Thus, the school community becomes more likely to recognize the symptoms of the disease and the general measures for its control, in addition to identifying early cases of undiagnosed asthma in schools [11, 17, 19, 20, 24].

The possibility of identifying suggestive asthma symptoms in the school environment is one of the biggest benefits of school-based interventions [11], although it is little explored in the studies analyzed [12, 17]. The underdiagnosis of asthma is responsible for high rates of undertreatment, exacerbating the illness and increasing school absenteeism among children and adolescents [36, 37]. Adequate knowledge of asthma and the early detection of suspected cases of asthma at school [9, 11], along with the due referral to the health service, can be a simple alternative to reduce the social, personal, and economic development of asthma in school-age individuals [11, 15].

Health outcomes could not be changed using only the improvement of knowledge among asthmatic patients. The extensive knowledge about the disease by asthmatics and nonasthmatics, perhaps, potentially benefits asthma management and morbidity control. In this review, only eight of the studies (47%) adopted knowledge as one of the analyzed outcomes. The studies evaluating asthma knowledge in their results showed that an increased knowledge of the disease can reduce morbidity indicators [18] and the emotional burden of the disease among asthmatic children and adolescents [19, 21] and increase the use of inhaled corticosteroids [16] with a better inhalation technique [31]. However, not all of the intervention models were sufficient in achieving these results. In this review, we found these results in interventions with educational group sessions that were led by health professionals and with a duration of, at maximum, 50 minutes [18, 19, 21, 31].

Although some of the studies analyzed showed reductions in hospitalization instances [14, 18, 29, 34], visits to the emergency department [14, 18, 22, 30], and the frequency and intensity of asthma symptoms [14, 16, 29, 33, 35], a high proportion of studies presented no reduction or analysis of these outcomes (47%). The information regarding the diagnosis of asthma, the severity and control of the disease, and the use of health services, for example, was reported by students and their parents [14, 16, 18, 22, 29, 30, 34, 35] and may underestimate the control of the disease. Legal restrictions on the permitted use of medications in class and the unpreparedness of school staff in the management of cases in schools may be factors that hinder access to appropriate care at the school [13, 15]. Hypothetically, these limitations may have influenced the reduction of the impact of interventions on morbidity indicators.

A reduction of school absenteeism [14, 18, 22, 29, 34] and an improvement in quality of life [14, 19, 21, 22, 30, 33, 34] were verified among asthmatic individuals who participated in the interventions. A high level of school absenteeism can cause great personal suffering, affect social and intellectual development, and even lead to long-term consequences, such as the loss of productivity and early retirement [4, 9, 38]. Thus, it becomes imperative to develop effective strategies to reduce these outcomes, such as the association of examples of school-based interventions with adequate medical care and the construction of protocols or guidelines for the prevention and control of asthma symptoms aimed by the school community [15].

Only one study showed an increase in the frequency of use of the action plan among the asthmatic students [14]. In this study, greater adherence to the action plan was linked to the combination of educational asthma interventions in schools with a medical follow-up of asthmatic adolescents who participated in the school intervention. This intervention model may have been the factor that was responsible for greater compliance with the intervention plan. Corroborating this result, other studies address the direct relationship between education programs, adequate medical care, and the success of interventions [13, 15]. However, in some low- or middle-average-income countries, there are no legal provisions regulating the obligation of health professionals in schools. Thus, the creation of strategies that allow for a partnership between schools and the healthcare service is necessary [15].

In this context, health professionals can not only act in areas of prevention, diagnosis, treatment, and education but also raise awareness and form intervention groups in schools [39]. These professionals should prioritize the maintenance of healthy children so that these individuals reach adulthood without any adverse influences, such as poor diet, smoking, and physical inactivity, resulting from their childhood or adolescence. All of these behaviors can impact the exacerbation of chronic health conditions such as asthma and obesity and need to be avoided.

Guidelines and public policies establishing health and asthma programs in schools should be implemented. In Brazil, for example, we recognize the existence of the program* Health in Schools* which aims, among other objectives, to promote better health practices among children and adolescents [23] using the integration of population health-related content on the school curriculum [23]. Asthma has been overlooked in this program despite affecting 24.3% and 17.5% of Brazilians in the age groups of 6-7 years [3] and 13-14 [40] years, respectively.

The school is a productive environment for health promotion, and all of the studies analyzed in this review support this hypothesis. The school environment is recognized by guidelines and public policies as a favorable space for spreading a culture of prevention and the development of healthy behaviors among school-age individuals [9, 11, 24, 25, 41]. In this way, educational initiatives for health should be developed in schools to sensitize children, adolescents, families, and school staff to the adoption of healthy behaviors in the short, medium. and long term.

With school education, training for citizenship and new cultures should be the goal [42, 43]. Toward this end, it is proposed that issues, such as external health causes, violence, alcohol, tobacco, and other drugs and healthy eating [39], in addition to illnesses of public interest, make up the general content of each conventional discipline (Portuguese, mathematics, science, geography, etc.) [42]. This strategy could be feasible for dissemination and popularization of health information.

Although only 3 studies present methods applicable to low- or middle-income countries, all of the studies substantially increase the knowledge of asthma among individuals of school age, asthmatics [17, 19] or not [20]. This fact ensures that the popularization of knowledge about asthma in schools through curricular integration may be feasible due to it being an inexpensive and reproducible strategy. This insertion of content should be systematic and continuous, valuing noninclusion of new subjects in the curriculum [23, 44]. Furthermore, it should prioritize the use of innovative and attractive teaching techniques for students and teachers.

In Brazil, the authors of this review adapted a model developed in the USA [20] based on public policy and national and international guidelines. This model, which is a pioneering approach in our country, included topics on health and correlated with asthma in the school curriculum, for students, asthmatics and nonasthmatics, in subjects such as Portuguese, science, biology, and current affairs. It is an intervention conducted by sensitized teachers and supported by the school management who adopted teaching techniques that are considered attractive to children and adolescents. Preliminary results suggest that this intervention model substantially increases the knowledge of asthma of the whole school community and promotes a culture of prevention and safer attitudes in managing the disease. It is a model adapted to the regional needs of our country without additional human resources needed on the part of the school and is an additional strategy for the control of chronic health problems such as asthma [44].

Asthma education for disease control is recommended with the highest level of scientific evidence [45, 46]. Such education must be implemented in different areas, such as emergency departments, hospitals and basic health units, the health strategies of the family, households, and school environments. Asthma education should precede the public policies of access to health and availability of medications to treat the disease. In addition, the civil empowerment favored by public education promotes the requirement of better health conditions [10].

Although it is not an ideal scenario, in our country of Brazil, we have observed the evolution of public policies. Thus, access to treatment and services has been promoted for various health disorders, including asthma. In this context, the availability of asthma medication in our country has favored the creation and consolidation of programs for the control of asthma, and many of these programs have been successful [47]. These initiatives are isolated and not standardized for age or the degree of severity of the disease and include multidisciplinary monitoring and information on asthma in addition to distributing antiasthma inhaled medication [47, 48]. Additionally, maintenance and relief treatments have been made available for free for the most mild and moderate forms of asthma in pharmacies [48].

A complementary strategy for the dissemination of health knowledge may be the training of monitors in schools and multipliers in other community spaces [10]. In this review, the authors of one of the studies support the training of monitors of the same age for carrying out educational asthma interventions in schools with a positive impact on the knowledge of the disease and an improved inhalation technique [21]. It is necessary that this training of monitors and multipliers goes beyond the school premises and involves the participation of the surrounding community in educational asthma interventions because communities can act as potential multipliers of information of the disease [10, 23]. The training of trainers becomes yet another strategy that is likely to favor the reduction of asthma morbidity through the knowledge of measures to prevent and control the disease.

Distinct methodological limitations observed in studies with examples from different periods of follow-up research and inadequate school records of absence due to asthma should be considered, rather than the consolidated methods. Another limitation to note is the prevalence of individuals with a mild form of the disease who reported a minimal use of the health service. Homogeneity of statistical analyses was not observed in the included studies, as this is a systematic review of literature without meta-analyses. The application of questionnaires for methodological quality evaluation of the studies can be considered dispensable to avoid the relevant restrictions in the selection of studies. This is justified due to the peculiarity of methodological aspects concerned to educational asthma interventions. The authors consider that the inclusion of few studies with lower methodological rigor did not change the final results and conclusions for this review.

## 5. Conclusion

Different models of educational asthma interventions carried out in schools can improve knowledge of the disease among asthmatic and nonasthmatic students, parents, caregivers, and school staff. The reduced impact of these interventions on morbidity indicators was also observed in this review between diagnosed asthmatic students. We identified models of educational asthma interventions in low- and middle-income countries, which are regions that concentrate the highest proportion of asthmatics. These educational activities, targeted to whole school community and not restricted to only asthmatics, could be a strategy for the control of chronic diseases such as asthma.

## Figures and Tables

**Table 1:** Description of the 17 educational asthma interventions.

Main author	Location	Length of the study	Target audience	Interval between evaluations	Number of sessions	Duration of each session	Approach	Outcomes evaluated			Hospitalization	School absenteeism	Use of relief medication/inhalation technique
								Knowledge	Quality of life	Visit to the emergency department			
Butz [16]	Baltimore, Maryland, USA	10 months	201 asthmatic individuals, 6–12 years old, with their parents/caregivers	10 months	2 (students) and 1 (parent or caregiver)	120 min	Individual and group	X	X	X	X	—	X

Patterson [28]	Belfast, Northern Ireland	4 months	173 asthmatic individuals, 7–11 years old	8 weeks	8	*∗*	Group	—	X	—	—	—	X

Cicutto [22]	Toronto, Canada	12 months	239 asthmatics, 6–11 years old, with their parents	2-3 months	6	50–60 min	Group	—	X	X	—	X	—

Clarck [34]	Beijing, China	12 months	639 asthmatic individuals, 7–11 years old, with their parents	12 months	5	*∗*	Group	—	X	X	X	X	—

Gerald [17]	Alabama, USA	5 years	610 asthmatic individuals, 8–11 years old. General approach to nonasthmatics (no outcomes evaluated)	Variation among the schools. The objective was to complete 6 sessions	6	30 min	Group	X	—	X	X	X	—

Levy [18]	Memphis, Tennessee, USA	24 months	243 asthmatic individuals (first year) and 210 asthmatic individuals (second year), 6–10 years old	12 months	6	40 min	Group	X	—	X	X	X	—

Joseph [29]	Detroit, Michigan, USA	12 months	314 individuals with an average age of 15.3, with symptoms of asthma	12 months	4	30 min	Individual	—	X	X	X	X	X

Bruzzese [35]	New York, USA	2 months	23 asthmatic individuals, with an average age of 12.9, and 21 parents	2 months	6 (students) and 5 (parents)	75 min (students) and 90 min (parents)	Group	—	—	—	—	—	X

Kintner [19]	Michigan, USA	3 months	66 asthmatic individuals, with an average age of 10.1, and their caregivers	3 months	10	50 min	Group	X	X	—	—	—	—

Clark [12]	Detroit, Michigan, USA	24 months	1292 individuals, 10–13 years old, with symptoms of asthma	12 months	6	90 min	Group	—	X	—	—	—	—

McGhan [30]	Edmonton, Alberta, Canada	12 months	266 asthmatic individuals, 6–13 years old	6 months	6	45–60 min	Group	—	X	X	—	X	X

Bruzzese [14]	New York, USA	12 months	345 asthmatic individuals, with an average age of 15.1	6 months	8	45–60 min	Individual and group	—	X	X	X	X	X

Mosnaim [31]	Chicago, USA	1 month	536 asthmatic individuals, with an average age of 13–16 years old	1 month	4	45 min	Group	X	—	—	—	—	X

Pike [20]	St. Louis, USA	12 months	236 asthmatic and nonasthmatic individuals	12 months	15	*∗*	Group	X	—	—	—	—	—

Al-sheyab [21]	Jordan, Middle East	3 months	244 asthmatic individuals	3 months	3	30 min	Group	X	X	—	—	—	—

Bowen [32]	Newark, New Jersey, USA	1 month and 2 weeks	30 asthmatic individuals, 8–12 years old	3 weeks	3	90 min	Group	X	—	—	—	—	—

Joseph [33]	Detroit, Michigan, USA	12 months	422 asthmatic individuals, with an average age of 15.6	6 months	4	15–30 min	Group/individual	—	X	X	X	X	—

Note: ^*∗*^not detailed.

The age of participants of the selected studies in this review was limited from 10 to 19 years old considering that the World Health Organization defines this period of life as adolescence.

**Table 2:** Educational asthma interventions carried out in the school environment and their achieved outcomes.

Author/year	Intervention	Conduction of the intervention	Main outcomes					
			Knowledge of asthma	Quality of life	Visits to the emergency department	Hospitalization	School absenteeism	Use of medications
Butz et al. (2005) [16]	Educational asthma intervention, for students, with the use of coloring books, inhalers, and peak flow meters Workshop on asthma for the parents or caregivers	Health educators/nurses	*Students* ^*∗*^: IG, $\overset{-}{x}$ = 10.4 (after), versus CG, $\overset{-}{x}$ = 9.9 (*p* = 0.18) *Parents* ^*∗∗*^: IG, $\overset{-}{x}$ = 17.5, versus CG, $\overset{-}{x}$ = 16.3 (*p* = 0.0004)	There was no change in quality of life (*p* > 0.05)	IG, 13.4%, versus CG, 18% (*p* = 0.34)	IG, 3.6%, versus CG, 5.6% (*p* = 0.62)	—	*Use of maintenance medication*: IG, 52.7%, versus CG, 62.9% (*p* = 0.05)

Patterson et al. (2005) [28]	Intervention based on the theoretical model *Predisposing, Reinforcing and Enabling Causes in Educational Diagnosis and Evaluation (PRECEDE*)	Nurses	—	IG, $\overset{-}{x}$ ± SD = 0.30 ± 1.19, versus CG, $\overset{-}{x}$ ± SD = 0.23 ± 0.98 (*p* = 0.32)	—	—	—	Correct inhalation technique: IG, 56%, versus CG, 15% (*p* < 0.001)

Cicutto et al. (2005) [22]	Intervention based on the program *Roaring Adventures of Puff (RAP)*	Health educators/asthma certified	—	IG, $\overset{-}{x}$ ± SD = 5.5 ± 1.4, versus CG, $\overset{-}{x}$ ± SD = 5.0 ± 1.4 (*p* < 0.05)	IG, $\overset{-}{x}$ ± SD = 1.7 ± 1.9, versus CG, $\overset{-}{x}$ ± SD = 2.5 ± 2.5 (*p* < 0.01)	—	IG, $\overset{-}{x}$ ± SD = 3.0 ± 4.4, versus CG, $\overset{-}{x}$ ± SD = 4.3 ± 5.7 (*p* < 0.05)	—

Clark et al. (2005) [34]	Adaptation of the curricular educational program *Open Airways for Schools* for students with asthma and their parents	Health educators	—	Rural area: IG, $\overset{-}{x}$ = −0.132, versus CG, $\overset{-}{x}$ = −0.577 (*p* = 0.04) Industrial area: IG, $\overset{-}{x}$ = −0.628, versus CG, $\overset{-}{x}$ = −0.340 (*p* = 0.001)	There was no difference between the IG and the CG (*p* > 0.05)	Schools in industrial areas: larger reduction in hospitalization instances (OR: 1.96, *p* = 0.05)	CG, $\overset{-}{x}$ = −0.55, versus IG, $\overset{-}{x}$ = −0.32 (*p* = 0.02)	—

Gerald et al. (2006) [17]	Curricular educational programs *Open Airways for Schools* (for students with asthma; main focus), *Managing Asthma: A Guide for Schools* (for school staff), and *Asthma Awareness: A Curriculum for the Elementary School Classroom *(for all students)	Health professionals	Difference of 3 points in the IG (0.23–4.09) (*p* < 0.001)	—	IG, $\overset{-}{x}$ ± SD = 0.09 ± 0.28, versus CG, $\overset{-}{x}$ ± SD = 0.10 ± 0.31 (*p* > 0.05)	IG, $\overset{-}{x}$ ± SD = 0.04 ± 0.19,versus CG, $\overset{-}{x}$ ± SD = 0.02 ± 0.14 (*p* > 0.05)	IG, $\overset{-}{x}$ ± SD = 3.88 ± 3.5, versus CG, $\overset{-}{x}$ ± SD = 3.21 ± 3.2 (*p* > 0.05)	—

Levy et al. (2006) [18]	Intervention based on curricular program *Open Airways for Schools*	Nurses	IG: 40% (before) versus 87% (after) (*p* < 0.0001) Increased knowledge also observed among the parents of the IG (*p* < 0.01)	—	IG, $\overset{-}{x}$ ± SD = 1.36 ± 0.49, versus CG, $\overset{-}{x}$ ± SD = 1.59 ± 1.0 (*p* < 0.001)	IG, $\overset{-}{x}$ ± SD = 0.18 ± 0.73, versus CG, $\overset{-}{x}$ ± SD = 0.45 ± 1.06 (*p* < 0.05)	IG, $\overset{-}{x}$ ± SD = 4.38, versus CG, $\overset{-}{x}$ ± SD = 8.18 (*p* < 0.05)	—

Joseph et al. (2007) [29]	Online and interactive program based on *National Asthma Education and Prevention Programs*	Educational software on asthma	—	There was no difference between the IG and the CG (*p* = 0.35)	There was no difference between the IG and the CG (*p* = 0.08)	IG: < hospitalization instances (*p* = 0.01)	IG < school absenteeism (*p* = 0.006)	*Comprehension of relief medication*: IG, 38.8%, versus CG, 32.2% (*p* = 0.01)

Bruzzese et al. (2008) [35]	Intervention based on curricular programs *Open Airways for Schools* and *Asthma Self-Management for Adolescents* (ASMA)	Nurses	—	—	—	—	—	IG, $\overset{-}{x}$ ± SD = 3.0 ± 0.9, versus CG, $\overset{-}{x}$ ± SD = 2.2 ± 0.8 (*p* < 0.05)

Kintner and Sikorskii (2009) [19]	Interactive program, using educational books and curriculum SHARP (Staying Healthy-Asthma Responsible and Prepared)	Health professionals	IG, $\overset{-}{x}$ ± SD = 10.18 ± 0.43, versus CG, $\overset{-}{x}$ ± SD = 7.96 ± 0.47 (*p* < 0.01)	IG, $\overset{-}{x}$ ± SD = 2.13 ± 0.10, versus CG, $\overset{-}{x}$ ± SD = 1.70 ± 0.11 (*p* < 0.01)	—	—	—	—

Clark et al. (2010) [12]	Intervention composed of two programs: (i) adaptation of *Open Airways for schools* and (ii) orientation on self-management with participation of classmates	Health studies undergraduates, community leaders, and students of previously trained primary education	—	There was no difference between the IG and the CG (*p* > 0.005)	—	—	—	—

McGhan et al. (2010) [30]	Program based on *Roaring Adventures of Puff (RAP)*	Health professionals	—	IG, $\overset{-}{x}$ = 5.9, versus CG, $\overset{-}{x}$ = 4.9 (*p* < 0.05)	IG, $\overset{-}{x}$ = 0.2, versus CG, $\overset{-}{x}$ = 0.072 (*p* < 0.05)	—	IG, $\overset{-}{x}$ = 4.0, versus CG, $\overset{-}{x}$ = 2.5 (*p* > 0.05)	Use <3 puffs B2 of short action: IG, 77.4%, versus CG, 70% (*p* < 0.05)

Bruzzese et al. (2011) [14]	Educational and clinical intervention incorporated into school hours: ASMA (Asthma Self-Management for Adolescents)	Nurses and medical specialists	—	Increase in quality of life in the IG (*p* = 0.0045)	IG: $\overset{-}{x}$ ± SD = 0.64 ± 1.51 CG: $\overset{-}{x}$ ± SD = 1.46 ± 3.69 (*p* < 0.0001)	IG: $\overset{-}{x}$ ± SD = 0.05 ± 0.30 CG: $\overset{-}{x}$ ± SD = 0.24 ± 1.18 (*p* = 0.0042)	IG: $\overset{-}{x}$ ± SD = 0.43 ± 0.69 CG: $\overset{-}{x}$ ± SD 0.78 ± 1.09 (*p* = 0.004)	IG: $\overset{-}{x}$ ± SD = 46.04 ± 76 CG: $\overset{-}{x}$ ± SD 43.66 ± 64 (*p* = 0.47)

Mosnaim et al. (2011) [31]	Educational curricular intervention on asthma using focus groups and technical inhaler training	Nurses, medical specialists, and health educators	Adolescents increase of 0.85 points (*p* < 0.0001)	—	—	—	—	Better use of inhalers by the adolescents (IG), *p* < 0.0001

Pike et al. (2011) [20]	Curricular intervention integrating the theme of asthma in 3 primary education subjects	Previously trained primary education teachers	Before versus after test >33% in the IG (*p* < 0.001)	—	—	—	—	—

Al-sheyab et al. (2012) [21]	“Triple A” intervention aimed at the training of peers	Bilingual nurses trained by the people responsible for “Triple A”	IG, $\overset{-}{x}$ ± SD = 7.14 ± 0.20, versus CG, $\overset{-}{x}$ ± SD = 5.52 ± 0.20 (*p* = 0.03)	IG, $\overset{-}{x}$ ± SD = 5.42 ± 0.14, versus CG, $\overset{-}{x}$ ± SD = 4.07 ± 0.14 (*p* = 0.02)	—	—	—	—

Bowen (2012) [32]	Adaptation of the program *Open Airways for Schools* integrated into the school curriculum and school hours	Health professionals	IG (70–90%) versus CG (50%) (*F* = 19.028, *p* < 0.001)	—	—	—	—	—

Joseph et al. (2013) [33]	Online interactive program *Puff City*	Health professionals/educational software on asthma	—	IG, $\overset{-}{x}$ ± SD = 5.3 ± 7.4, versus CG, $\overset{-}{x}$ ± SD = 7.1 ± 7.6 (*p* = 0.025)	IG: $\overset{-}{x}$ ± SD = 1.5 ± 3.4 versus 1.7 ± 3.7 (*p* = 0.95)	IG, $\overset{-}{x}$ ± SD = 0.3 ± 0.8, versus CG, $\overset{-}{x}$ ± SD = 0.5 ± 1.1 (*p* = 0.47)	—	—

*Note.* Open Airways for Schools is a program that educates and empowers children in the self-management of asthma through an interactive approach. Roaring Adventures of Puff (RAP) is an educational program on asthma for children and health professionals, with a view to addressing the lack of educational activities on asthma for the self-management of the disease. IG: intervention group; CG: control group.

The age of participants of the selected studies in this review was limited from 10 to 19 years old considering that the World Health Organization defines this period of life as adolescence.

^*∗*^Children grades 3–5.

^*∗∗*^Parent asthma knowledge scores.
